# Plasma Metabolites Link Non‐Communicable Diseases to Increased White Matter Hyperintensities

**DOI:** 10.1111/cns.70507

**Published:** 2025-07-28

**Authors:** Nan Wang, Baoshan Qiu, Weiqi Chen, Yuesong Pan, Yilong Wang

**Affiliations:** ^1^ Department of Neurology Beijing Tiantan Hospital, Capital Medical University Beijing China; ^2^ China National Clinical Research Center for Neurological Diseases Beijing China

**Keywords:** metabolites, multimorbidity, non‐communicable diseases, white matter hyperintensities

## Abstract

**Aims:**

Cerebral small vessel disease (CSVD) and non‐communicable diseases (NCDs) are major global health burdens. White matter hyperintensities (WMH) are a key imaging feature of CSVD, but the relationship between WMH and NCDs, especially the role of plasma metabolites in this association, remains unclear. This study aims to elucidate this link.

**Methods:**

This study included participants with WMH from the UK Biobank cohort and examined the prevalence of 29 common NCDs in this population. General linear regression was used to analyze the association between NCDs and WMH. Propensity score matching and elastic net regression identified plasma metabolites associated with NCDs. Mediation analysis was conducted to explore the role of these metabolites in the association between NCDs and WMH.

**Results:**

A total of 44,630 participants were included, of whom 47.0% were male. Approximately one‐third of the participants had NCDs, with the most common being hypertension, dyslipidemia, and asthma. Compared to those without NCDs, the WMH volume in individuals with one or more comorbid NCDs was significantly increased by 18.43% to 68.15%. During a median follow‐up of 9.5 years, individuals with hypertension, obstructive sleep apnea, and hypertension combined with coronary ischemic heart disease had significantly larger WMH volumes compared to those without NCDs, with increases of 30.81%, 36.44%, and 36.75%, respectively. Further analysis revealed that plasma metabolites associated with NCDs mediated this risk.

**Conclusions:**

This study elucidated the association between WMH and NCDs, showing that common NCDs significantly increase WMH volume. Plasma metabolites associated with NCDs mediate this risk. This provides new insights into preventing WMH progression in individuals.

## Graphical abstract

Approximately one‐third of individuals with white matter hyperintensities (WMH) have at least one common non‐communicable disease (NCDs). Individuals with NCDs have a larger WMH volume, with NCD‐related plasma metabolites partially contributing to this increase.

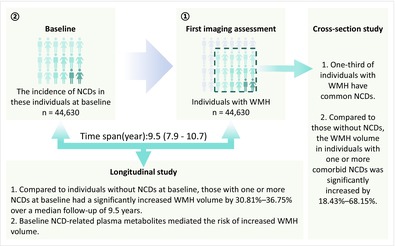

## 1 Introduction

White matter hyperintensities (WMH) are a key imaging feature of cerebral small vessel disease (CSVD). Multiple reports, including the Helsinki Aging Study and the Rotterdam Scan Study, have shown that the prevalence of any WMH in the general population ranges from 39% to 96% [1, 2]. Reports from China indicate that among stroke‐free community‐dwelling individuals aged 35 to 80, the prevalence of WMH ranges from 65.4% to 72.1% [3]. Importantly, a meta‐analysis has shown that WMH significantly increases the risk of stroke, dementia, and mortality [4]. This poses a major threat to global public health.

Currently, multimorbidity, defined as the coexistence of two or more non‐communicable diseases (NCDs), is a critical topic. Globally, the number of individuals with multiple NCDs is expected to rise significantly, posing challenges to research that focuses on single diseases [5]. Importantly, many common pathological mechanisms may exist between different diseases, influencing each other. Claudia Langenberg identified 420 metabolites shared between at least two NCDs, accounting for 65.5% of all disease‐associated metabolites [6]. For example, cerebrovascular diseases share metabolic features—amino acids, lipids, and other metabolites—with cardiovascular and respiratory diseases [6]. Interestingly, common NCDs such as hypertension and obstructive sleep apnea (OSA) are major risk factors for WMH [7, 8, 9]. This suggests that some NCD‐related metabolites may contribute to the development or progression of WMH. Notably, several previous studies have reported associations between various circulating metabolites and WMH [10, 11]. However, no studies have specifically examined the prevalence of NCDs in individuals with WMH, and in particular, the potential role of metabolites in the association between WMH and NCDs remains unclear.

Based on these issues, we aim to describe the comorbidity of NCDs in individuals with WMH, analyze the impact of common NCDs on WMH, and explore the role of NCD‐related plasma metabolites in this relationship.

## 2 Methods

### 2.1 Study Design

**FIGURE 1:**
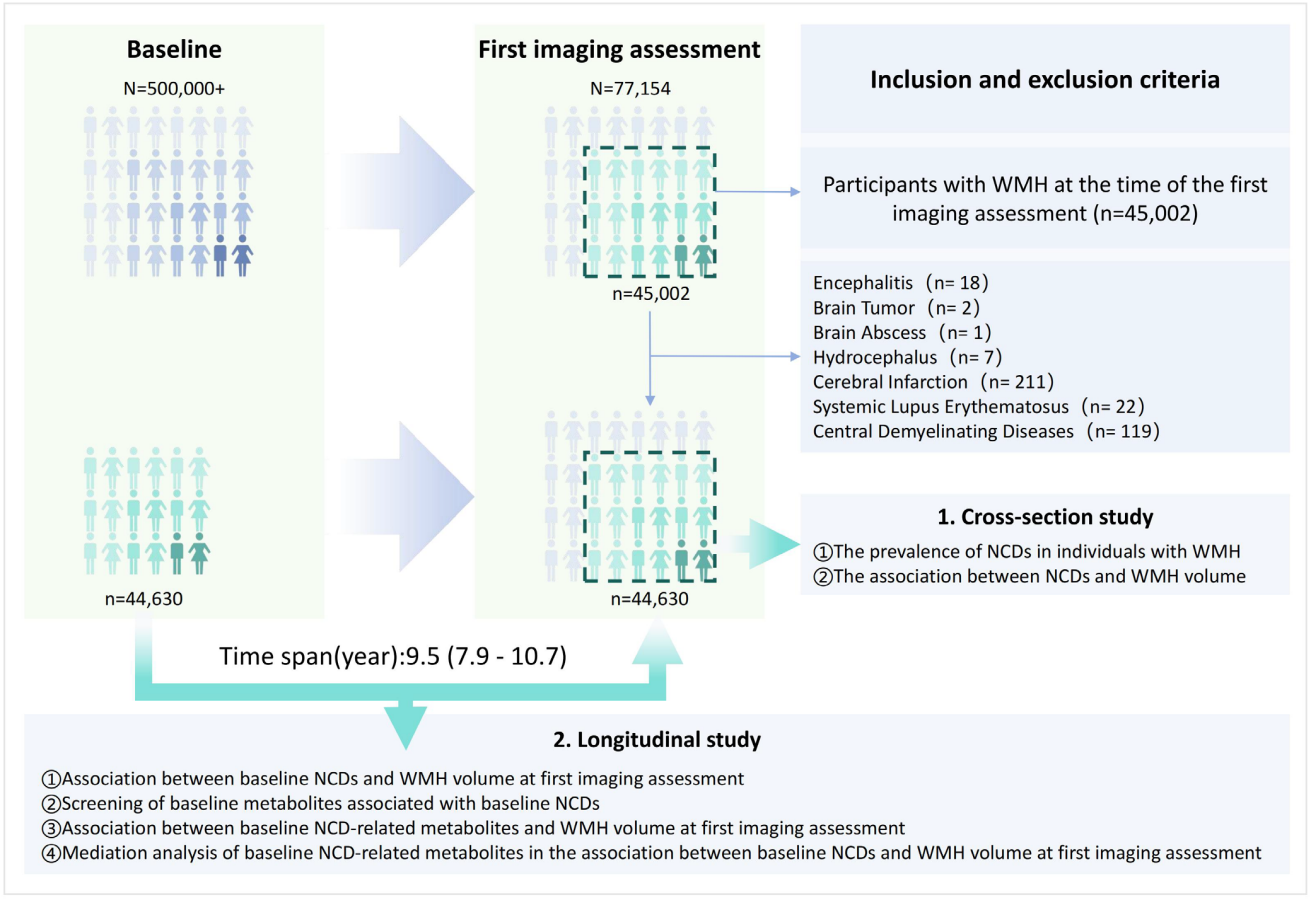
Study design and process. NCDs, non‐communicable diseases; WMH, white matter hyperintensities.

This study combines cross‐sectional and longitudinal cohort designs. First, we explored the comorbidity of NCDs in individuals with WMH at the time of the first imaging assessment in the UK Biobank cohort. Next, we analyzed the association between NCDs and WMH volume. We then reviewed the prevalence of NCDs at baseline and examined the relationship between baseline NCDs and WMH volume at the time of the first imaging assessment, with a median follow‐up period of 9.5 years. We screened for baseline NCD‐related metabolites and explored their association with WMH volume at the time of the first imaging assessment, further assessing their mediating effect in the relationship between baseline NCDs and WMH volume at first imaging assessment (Figure 1).

### 2.2 Participants

The study population was drawn from the UK Biobank, a globally recognized cohort that recruited over 500,000 voluntary participants between 2006 and 2010. Its design and demographics have been extensively documented in high‐quality studies. Notably, the first imaging assessments were conducted from 2014 to 2019.

This study included participants with WMH at the time of the first imaging assessment. Individuals diagnosed with central nervous system demyelinating diseases, such as hydrocephalus, various encephalitides, brain tumors, brain abscesses, systemic lupus erythematosus, cerebrovascular infarction, or multiple sclerosis, based on ICD‐10 criteria, were excluded (Table S1).

The study ethics of the UK Biobank have been approved by the National Health and Social Care Information Governance and the NHS North West Multi‐Center Research Ethics Committee. All participants provided informed consent via electronic signature at the time of recruitment. This study was conducted under UK Biobank application number 135114.

### 2.3 Brain Magnetic Resonance Imaging and WMH Volume

Some participants in the UK Biobank cohort underwent their initial imaging assessment in 2014. The scanner used is a standard Siemens Skyra 3 T running VD13A SP4 (as of October 2015), equipped with a standard Siemens 32‐channel RF receive head coil (http://biobank.ctsu.ox.ac.uk/showcase/showcase/docs/brain_mri.pdf). It is well‐known that the UK Biobank (UKB) data includes six MRI modalities: T1‐weighted and T2‐weighted fluid‐attenuated inversion recovery (T2‐FLAIR) structural images, susceptibility‐weighted MRI, diffusion MRI, task‐based functional MRI, and resting‐state functional MRI [12]. The total volume of WMH is estimated to generate additional imaging‐derived phenotypes. This process primarily utilizes T2‐FLAIR data, along with T1 data, and lesion segmentation is automatically performed using the BIANCA tool [13].

### 2.4 Blood Sample Collection and Metabolite Measurement

The UK Biobank team collected venous blood samples at different time points, which were stored in an ultra‐low temperature freezing system for future use. The metabolite markers involved in this study primarily originate from venous plasma collected at baseline. Upon arrival from the UK Biobank laboratory, the samples were stored in Nightingale Health's −80°C freezers. Before metabolite extraction, frozen samples were slowly thawed at 4°C. Nightingale Health Plc. has completed biomarker profiling of baseline plasma samples for all 500,000 UK Biobank participants, with its NMR biomarker platform detailed previously [14, 15]. A total of 249 metabolic parameters were assessed using high‐throughput NMR spectroscopy, including 168 absolute levels and 81 ratio values. The biomarkers span multiple metabolic pathways, including lipoprotein lipids in 14 subclasses, fatty acids and fatty acid compositions, as well as various low‐molecular‐weight metabolites, such as amino acids, ketone bodies, and glycolysis metabolites, all quantified in molar concentration units (Table S2). For the 14 lipoprotein subclasses, lipid concentration and composition were measured based on triglycerides, phospholipids, total cholesterol, cholesterol esters, and free cholesterol, as well as the total lipid concentration within each subclass. The majority of biomarker measurements are reported in absolute concentration units (mmol/L).

### 2.5 Defining NCD Variables

To date, there is no universally accepted or specific list of NCDs, and different studies have selected various types of diseases for investigation. A report from the University of Cambridge's Epidemiology Unit included 27 NCDs, while a research team from China incorporated 57 NCDs in their study [6, 16]. In an effort to understand the specific list of non‐communicable diseases, Bruce Guthrie conducted a detailed review of 566 studies involving multimorbidity (the coexistence of two NCDs), finding that 452 studies (79.9%) included 67 NCDs [17]. A study group using the Delphi consensus method distributed questionnaires and found that 35 NCDs were identified by professionals as conditions that should be considered for inclusion in a reference list of NCDs [18]. In this study, 29 common NCDs were selected based on the research objectives and relevant expert knowledge. The diseases include hypertension, heart failure, atrial fibrillation and flutter (AFF), cardiomyopathy, chronic ischemic heart disease (CIHD), dyslipidemia, type 2 diabetes mellitus (T2DM), hypothyroidism, hyperthyroidism, obesity, chronic gastritis, constipation, liver failure, chronic hepatitis, liver fibrosis and cirrhosis, fatty liver, intestinal malabsorption, chronic renal failure, chronic nephritis, nephrotic syndrome, nutritional anemia, chronic obstructive pulmonary disease (COPD), asthma, emphysema, respiratory failure, nonorganic headache, obstructive sleep apnea, anxiety disorder, and depressive disorder. These conditions were determined based on ICD‐10 diagnostic codes in the UK Biobank, as detailed in the Table S3.

### 2.6 Covariates

Covariates include sex, age, race, systolic blood pressure, diastolic blood pressure, BMI, smoking status, alcohol consumption, sleep duration, physical activity, household income, and educational level.

### 2.7 Statistical Analysis

Continuous variables were expressed as means ± standard deviation (SD) or medians (inter‐quartile range, IQR). Continuous variables were assessed for normality using the Kolmogorov–Smirnov test, and group comparisons were performed using either the independent *t*‐test or the Mann–Whitney U test. Categorical variables were represented as frequencies (percentages), and significance was evaluated with the χ^2^ test or Fisher's exact test. Upset analysis was applied to explore the distribution of NCDs across WMH volume quartiles. General linear regression was used to examine the relationship between NCDs and WMH volume. WMH volume was log‐transformed to correct for skewed distributions. Elastic net regression was employed to identify NCD‐associated metabolite markers after propensity score matching for sex, age, and BMI. General linear regression was used to examine the association between NCD‐associated metabolites and WMH volume. To account for multiple comparisons, *p*‐values were adjusted using the Benjamini–Hochberg method for false discovery rate (FDR) correction. Mediation analysis was conducted to evaluate the mediating role of NCD‐associated metabolites in the relationship between NCDs and WMH volume. The mediator and outcome models were adjusted for covariates. Indirect, direct, and total effects were estimated using nonparametric bootstrapping (1000 simulations). Prior to selection and association analyses, missing values in the NMR metabolomics data from the UK Biobank were imputed using median imputation, and all variables were standardized (mean centered to 0, standard deviation set to 1), without outlier detection. We conducted the analyses using three models: the first model was unadjusted, the second model was adjusted for sex and age, and the third model was fully adjusted for sex, age, systolic and diastolic blood pressure, BMI, smoking status, alcohol consumption, race, income, education level, sleep duration, and physical activity.

All data analyses were performed in R version 4.4.0 using the relevant packages, and data visualization was completed in R version 4.4.0 and GraphPad Prism 9. Statistical significance was defined as a two‐tailed *p*‐value < 0.05 or *p* FDR < 0.05.

## 3 Results

### 3.1 Characteristics of Participants at Baseline and First Imaging Assessment

This study included a total of 44,630 individuals with WMH at first imaging assessment, of whom 47.0% were male and 96.7% were of white ethnicity. The baseline mean age was 55.0 ± 7.54 years, and the average age at the first imaging assessment was 64.3 ± 7.74 years, with a follow‐up period of 9.5 (7.9–10.7) years. The mean BMI at baseline and at the first imaging assessment was 26.6 ± 4.21 and 26.5 ± 4.38, respectively. The alcohol consumption rate exceeded 90% at both time points, while the smoking rates were 6.1% and 3.3%, respectively (Table 1).

**TABLE 1:** Characteristics of participants at baseline and first imaging assessment.

	Participants (*N* = 44,630)	
	Baseline	First imaging assessment
Male, *n* (%)	20,995 (47.0)	20,995 (47.0)
Age (year), Mean ± SD	55.0 ± 7.54	64.3 ± 7.74
Ethnicity^a^, *n* (%)		
White	43,170 (96.7)	43,170 (96.7)
Non‐White	1337 (3.0)	1337 (3.0)
SBP (mmHg), Mean ± SD	137 ± 18.7	142 ± 20.2
DBP (mmHg), Mean ± SD	81.3 ± 10.4	78.9 ± 10.7
BMI, Mean ± SD	26.6 ± 4.21	26.5 ± 4.38
Smoking status^a^, *n* (%)		
Never	27,143 (60.8)	27,738 (62.2)
Previous	14,653 (32.8)	14,955 (33.5)
Current	2736 (6.1)	1472 (3.3)
Alcohol drinker status^a^, *n* (%)		
Never	1084 (2.4)	1417 (3.2)
Previous	940 (2.1)	1473 (3.3)
Current	42,579 (95.4)	41,411 (92.8)
Household income (pounds)^a^, *n* (%)		
Less than 18,000	4649 (10.4)	4884 (10.9)
18,000–30,999	8835 (19.8)	10,851 (24.3)
31,000–51,999	12,161 (27.2)	12,217 (27.4)
52,000–100,000	11,443 (25.6)	9175 (20.6)
Greater than 100,000	3160 (7.1)	2956 (6.6)
Education^a^, *n* (%)		
College or university degree	20,400 (45.7)	20,400 (45.7)
No college or university degree	20,615 (46.2)	20,615 (46.2)
Sleep duration (hours/day), Mean ± SD	7.16 ± 1.00	7.14 ± 1.09
Physical activity (minutes/week), Median (IQR)	1733 (828.0–3276.0)	2194.0 (1125.0–3897.0)

*Note:* BMI, Body Mass Index; DBP, diastolic blood pressure; SBP, systolic blood pressure.

^a^ The results for “Do not know”, “Prefer not to answer”, and “Missing values” have been omitted.

### 3.2 Prevalence of NCDs in Individuals With WMH

**FIGURE 2:**
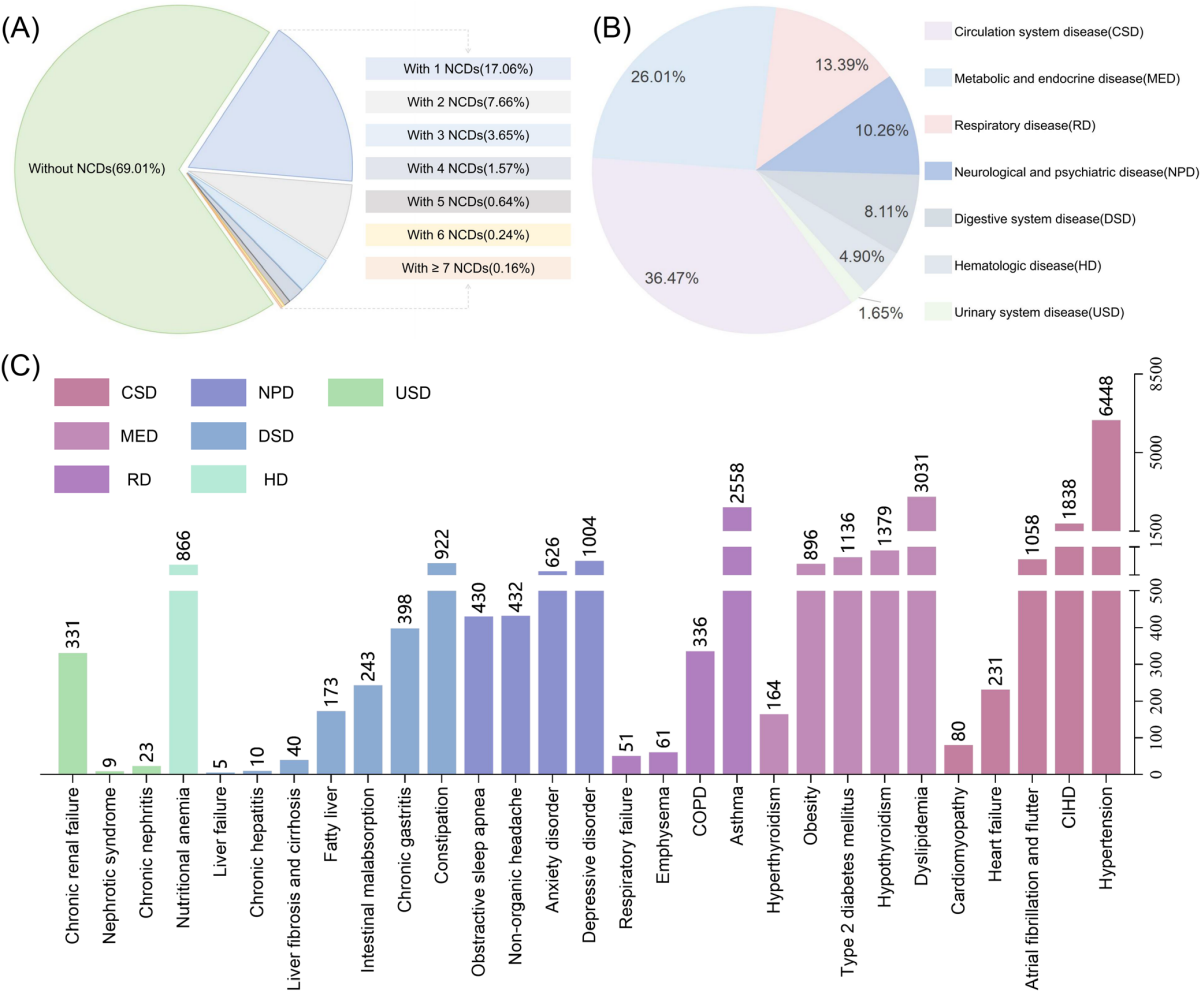
Prevalence of NCDs in individuals with WMH. (A) Number and proportion of individuals with NCDs. (B) Proportions of NCDs classified by disease system. (C) Distribution of the number of cases for 29 NCDs. CIHD, chronic ischemic heart disease; COPD, chronic obstructive pulmonary disease.

At the first imaging assessment, 30.99% of the population had at least one NCD, with 17.06% having only one NCD and 13.93% having two or more NCDs (Figure 2A). The most common comorbid NCDs were circulation system diseases, accounting for 36.47%, followed by metabolic and endocrine disorders at 26.01%, and respiratory diseases at 13.39%, together making up more than 75% of cases (Figure 2B). The top three comorbid NCDs were hypertension (6448 individuals), dyslipidemia (3031 individuals), and asthma (2558 individuals) (Figure 2C).

### 3.3 Distribution of NCDs in WMH Volume Quartiles

**FIGURE 3:**
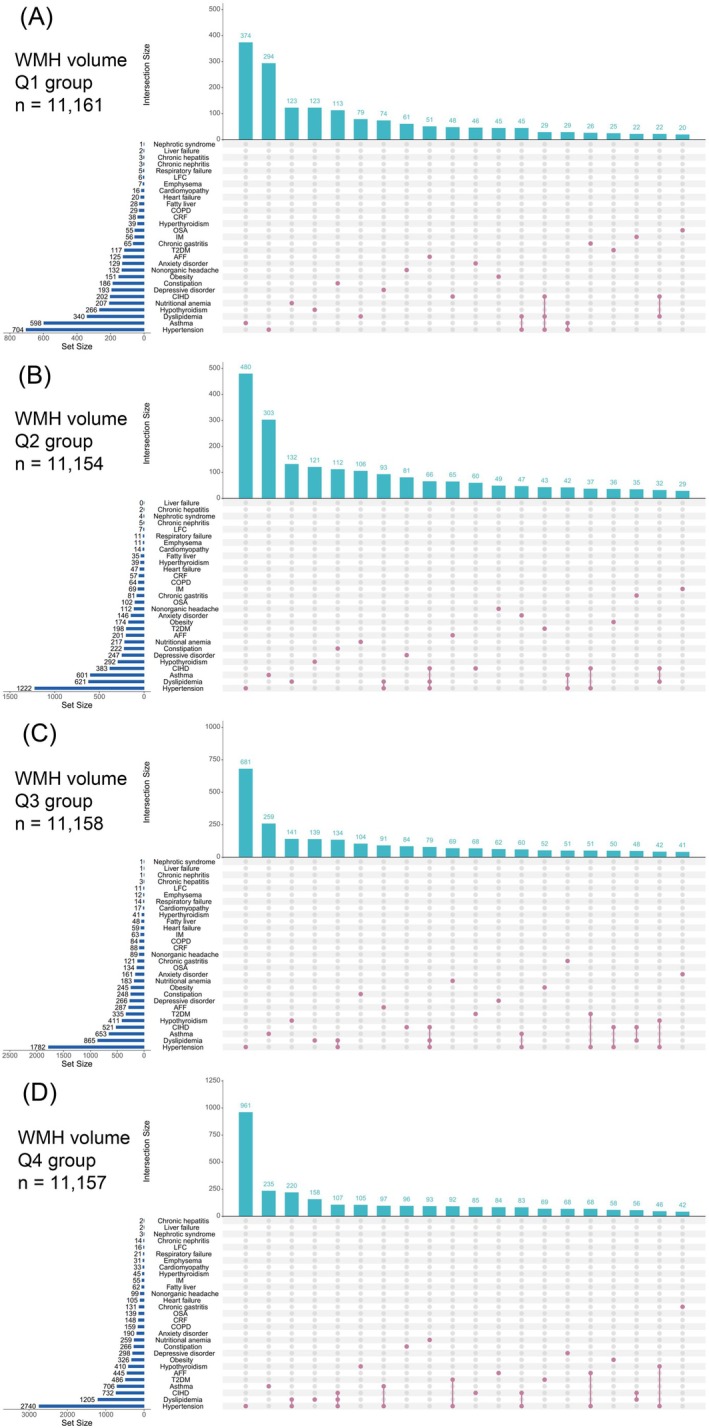
Distribution of NCDs in WMH volume quartiles. AFF, atrial fibrillation and flutter; CIHD, chronic ischemic heart disease; COPD, chronic obstructive pulmonary disease; CRF, chronic renal failure; IM, intestinal malabsorption; LFC, liver fibrosis and cirrhosis; OSA, obstructive sleep apnea; T2DM, Type 2 diabetes mellitus.

When stratified by WMH volume quartiles, as WMH volume increased, there was a rise in age, systolic and diastolic blood pressure, the proportion of males, the proportion of individuals with comorbid NCDs, and the proportion of those with circulation system diseases, metabolic, and endocrine disorders (Table S4). Among the WMH quartiles Q1 to Q3, the most common comorbidities were hypertension, dyslipidemia, and asthma. In Q4, CIHD replaced asthma as the most common comorbidity (Figure 3). The other dimension of the Figure shows the number of individuals who had only a specific condition or certain conditions (displaying the top 20 patterns by frequency). In the Q1 group, the largest number of individuals had only asthma, with 374 people; in the Q2–Q4 groups, the largest number had only hypertension, with 480, 681, and 961 people, respectively. Notably, as WMH volume increased, the patterns of comorbidity became more numerous and complex. In the Q1–Q4 groups, 4, 5, 7, and 8 distinct comorbidity patterns were observed, respectively, and the number of individuals with these comorbidities gradually increased. For example, the number of individuals with hypertension combined with dyslipidemia was 45, 93, 134, and 220 in Q1–Q4, respectively.

### 3.4 Association Between NCDs and WMH Volume

**FIGURE 4:**
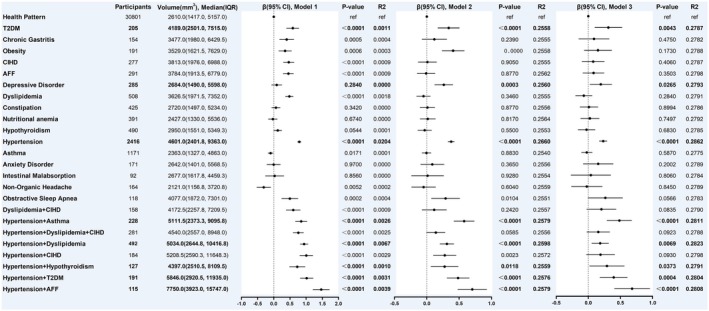
Association between NCDs and WMH volume at the first imaging assessment. Health pattern: Absence of any of the 29 NCDs; Model 1 was the unadjusted model; Model 2 was adjusted for age and sex; Model 3 was adjusted for age, sex, systolic blood pressure, diastolic blood pressure, BMI, smoking, alcohol consumption, ethnicity, income, education, sleep, and physical activity; AFF: atrial fibrillation and flutter; CIHD: chronic ischemic heart disease; T2DM: Type 2 diabetes mellitus.

Based on the results of the previous section in this study, we have summarized 24 common NCD patterns, including multimorbidity. In model 3, individuals with T2DM, depressive disorder, hypertension, hypertension combined with asthma, hypertension combined with dyslipidemia, hypertension combined with hypothyroidism, hypertension combined with T2DM, and hypertension combined with AFF had significantly higher WMH volumes compared to the health pattern (absence of any of the 29 NCDs), with increases of 31.22%, 19.44%, 23.05%, 48.25%, 18.43%, 28.97%, 39.38%, and 68.15%, respectively (all *p* < 0.05) (Figure 4).

**FIGURE 5:**
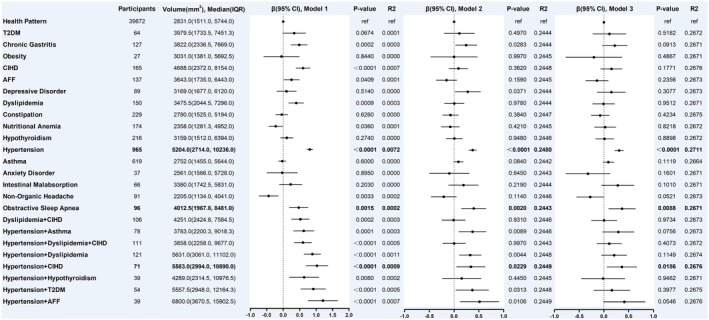
Association between baseline NCDs and WMH volume at first imaging assessment. Health pattern: Absence of any of the 29 NCDs; Model 1 was the unadjusted model; Model 2 was adjusted for age and sex; Model 3 was adjusted for age, sex, systolic blood pressure, diastolic blood pressure, BMI, smoking, alcohol consumption, ethnicity, income, education, sleep, and physical activity; AFF: atrial fibrillation and flutter; CIHD: chronic ischemic heart disease; T2DM: Type 2 diabetes mellitus.

To explore the long‐term effects of these comorbidity patterns on WMH, we reviewed the baseline NCD status of the target population and analyzed their association with WMH volume at the first imaging assessment. In model 3, individuals with baseline hypertension, OSA, and hypertension combined with CIHD had significantly higher WMH volumes at the 9.5 (7.9–10.7) years of follow‐up [median (IQR)] compared to the health pattern, with increases of 30.81%, 36.44%, and 36.75%, respectively (*p* < 0.05) (Figure 5).

### 3.5 Association Between NCD‐Associated Metabolites and WMH

**FIGURE 6:**
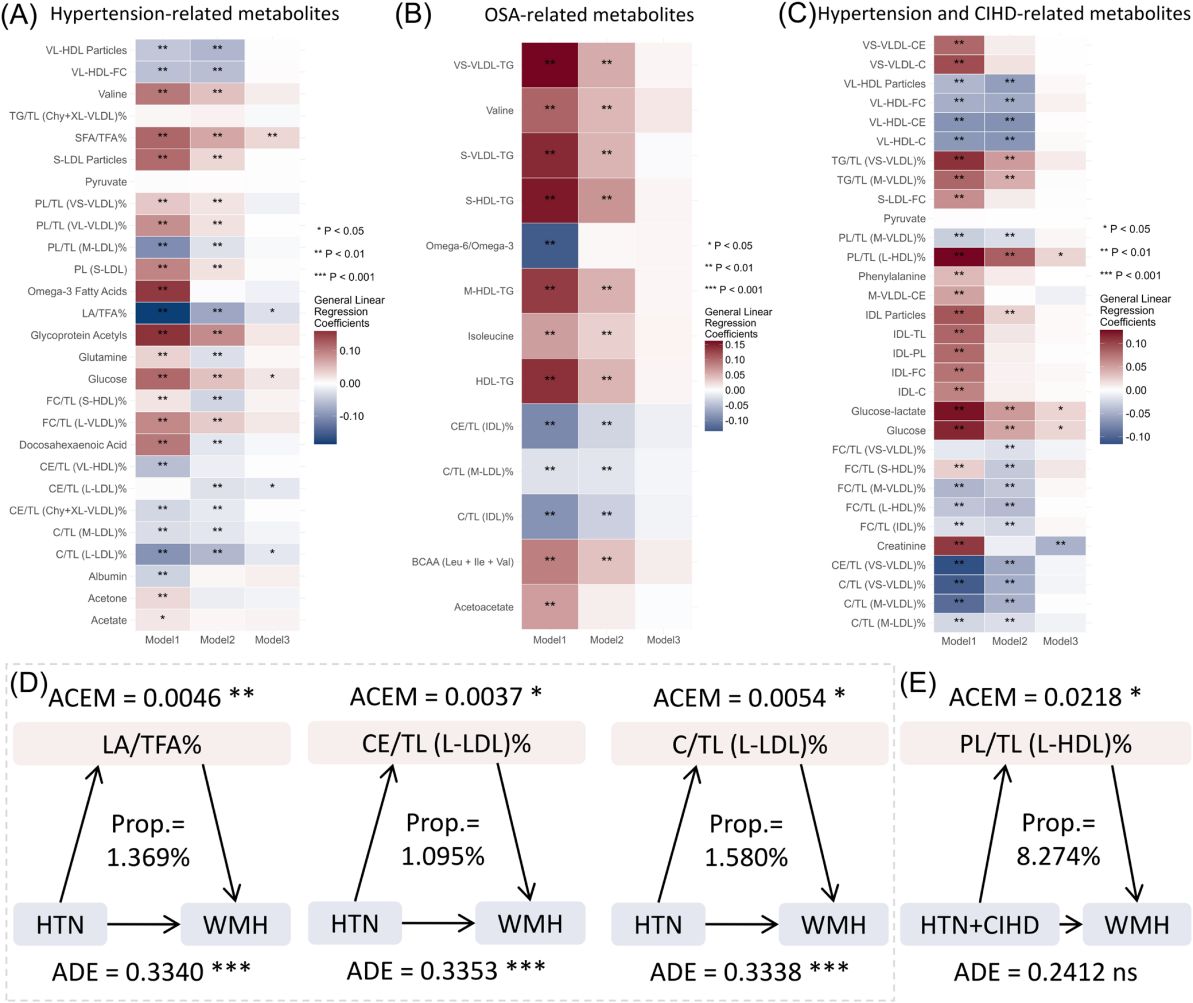
Association between baseline NCD‐related metabolites and WMH volume at first imaging assessment, and their mediating effect on the relationship between baseline NCDs and WMH volume at first imaging assessment. (A–C) Association analysis of metabolites related to hypertension, OSA, and hypertension combined with CIHD with WMH volume. (D, E) Mediating effect analysis of metabolites related to hypertension and hypertension combined with CIHD in the association with WMH volume. *p‐value < 0.05; **p FDR < 0.05; Model 1 was the unadjusted model; Model 2 was adjusted for age and sex; Model 3 was adjusted for age, sex, systolic blood pressure, diastolic blood pressure, BMI, smoking, alcohol consumption, ethnicity, income, education, sleep, and physical activity; All mediation analyses were conducted under Model 3. ACME, average causal mediation effect; ADE, average direct effect; C, cholesterol; CE, cholesteryl esters; CIHD, chronic ischemic heart disease; HTN, hypertension; LA, linoleic acid; L‐HDL, large high‐density lipoprotein; L‐LDL, large low‐density lipoprotein; PL, Phospholipids; TFA, total fatty acids; TL, total lipids. All metabolite abbreviations are listed in Table S5.

Based on sex, age, and BMI, a propensity score matching was performed in a 1:2 ratio with the health pattern people. Elastic net regression was then used to screen metabolites related to hypertension, OSA, and hypertension combined with CIHD. The optimal regularization parameter was set to shrink the coefficients of unimportant metabolites to zero while retaining important metabolites for differential metabolite selection (Figure S1A,B,D,E,G.H). Ultimately, 27, 13, and 31 metabolites related to hypertension, OSA, and hypertension combined with CIHD were identified, with bar plots displaying the coefficients of these metabolites in each respective model (Figure S1C,F,I). Further analysis examined the association of hypertension, OSA, and hypertension combined with CIHD‐related metabolites with WMH volume. In model 3, hypertension‐related metabolites such as SFA/TFA and glucose showed a significant positive correlation with WMH volume, while LA/TFA, CE/TL (L‐LDL), and C/TL (L‐LDL) showed a significant negative correlation with WMH volume (Figure 6A, all abbreviations of metabolic markers in Table S5). After adjustment in model 3, no significant correlation was found between OSA‐related metabolites and WMH volume (Figure 6B). For hypertension combined with CIHD in model 3, metabolites such as PL/TL (L‐HDL), glucose‐lactate, and glucose showed significant positive correlations with WMH volume, while creatinine showed a significant negative correlation with WMH volume (Figure 6C).

### 3.6 Mediation Analysis of Baseline NCD‐Associated Metabolites

To explore whether baseline NCD‐associated metabolites mediate the effect of baseline NCDs on WMH volume at first imaging assessment, we conducted mediation analysis. The results showed that LA/TFA, CE/TL (L‐LDL), and C/TL (L‐LDL) significantly mediated the effect of baseline hypertension on WMH volume at first imaging assessment, indicating partial mediation, with mediation effect proportions of 1.369%, 1.095%, and 1.580%, respectively (Figure 6D). PL/TL (L‐HDL) significantly mediated the effect of baseline hypertension combined with CIHD on WMH volume at first imaging assessment, indicating complete mediation, with a mediation effect proportion of 8.274% (Figure 6E). No significant mediation effects were observed for other metabolite markers (Figure S2).

## 4 Discussion

This study provides a comprehensive analysis of the relationship between common NCDs and WMH volume. We observed that WMH volume is influenced by both the pattern and number of coexisting NCDs. Longitudinal analysis further confirmed that conditions such as hypertension, as well as hypertension combined with chronic ischemic heart disease (CIHD), significantly increase WMH volume. Importantly, our findings extend previous research by identifying plasma metabolites associated with NCDs. These metabolites are not only linked to WMH volume but also mediate part of the association between NCDs and WMH. These results reveal potential metabolic pathways connecting NCDs to WMH burden and may inform future strategies for preventing WMH progression. In addition, we adjusted for key confounders such as household income and education. Prior studies have shown that individuals with lower socioeconomic status are more likely to engage in unhealthy lifestyles and less likely to adopt health‐promoting behaviors after NCDs diagnosis [19, 20]. Given the potential influence of socioeconomic factors on the relationship between NCDs and WMH burden, accounting for them enhances the validity and interpretability of our findings.

Our results show that among individuals with WMH, approximately one‐third have at least one common NCD. As WMH volume increases, the number of comorbid NCDs rises, and the pattern of multimorbidity becomes more complex. Consistent with previous studies, WMH volume significantly increases with the number of comorbidities [16]. This suggests that common pathological mechanisms may exist among NCDs, leading to exacerbated white matter damage. We observed that as WMH volume increases, the most prevalent conditions are hypertension, asthma, and hyperlipidemia. Elevated blood pressure can induce microvascular damage, characterized by smooth muscle cell loss in the tunica media, fibrohyaline deposition, luminal narrowing, and vessel wall thickening [21]. These microcirculatory changes may compromise cerebral perfusion and contribute to the development of white matter lesions, even in the pre‐hypertensive stage [22]. The primary pathophysiological mechanisms of asthma include chronic immune‐inflammatory responses and airway hyperreactivity. Increasingly, asthma patients are presenting with various neurological symptoms [23]. This may be due to the impact on brain structure and function through “lung‐brain axis” communication, where systemic inflammation could lead to disruption of the blood–brain barrier [24]. However, in our study, asthma was not found to have a significant direct impact on WMH volume. Interestingly, individuals with both hypertension and asthma exhibited greater WMH volume compared to those with hypertension alone. Their combined impact on WMH volume may exhibit a cumulative effect. In contrast, individuals with both hypertension and dyslipidemia had relatively smaller WMH volumes compared to those with hypertension alone. Dyslipidemia causes endothelial dysfunction, inflammation, and oxidative stress, but some studies have reported a negative correlation between dyslipidemia and WMH [25]. The types of lipids are highly complex. For instance, a study reported that lysophosphatidylcholines, hydroxysphingomyelins, and cholesteryl esters are likely protective factors for WMH, while low‐density lipoprotein (LDL), triglycerides, and phospholipids are more likely risk factors [10]. Additionally, diabetes may increase WMH by causing endothelial dysfunction and inflammatory responses [26, 27]. We observed that hypertension combined with diabetes could lead to a greater WMH volume. The relationship between the heart and brain has gained increasing attention, with left ventricular characteristics closely associated with the microstructure of white matter [28]. As seen in our results, hypertension combined with AFF, as well as hypertension combined with CIHD, significantly increased WMH volume. In summary, the presence of comorbid NCDs leads to a greater WMH burden, but the underlying connections remain unclear.

Multimorbidity has received increasing attention in real‐world settings. For instance, cardiometabolic diseases, including T2DM, heart disease, and stroke, can accelerate cognitive decline and increase the risk of dementia [29]. In fact, the coexistence of multiple diseases is not coincidental but represents a ‘disease cluster’ based on shared pathophysiological pathways, which has been confirmed at both genetic and metabolic levels [6, 30]. As research advances, increasing attention has been given to the brain‐lung, brain‐heart, and brain‐gut axes. These interconnected pathways further highlight the brain's close relationship with multiple organ systems and may help explain the biological basis of multimorbidity. Following the report by Claudia Langenberg's team, which demonstrated that a large number of metabolites are shared among NCDs [6]. Another team reported that plasma metabolomic profiles could serve as detection methods for multiple diseases. For example, amino acids, creatinine, and albumin are common predictors for both diabetes and all‐cause dementia [31]. In our study, glucose was significantly positively associated with WMH volume, hypertension, and hypertension combined with CIHD. The impact of glucose on white matter may involve inflammatory responses as well as damage to neurons and glial cells [32]. Elevated blood glucose is associated with the onset of primary hypertension [33]. Glucose metabolism is related to insulin release and insulin sensitivity, which in turn affects vascular smooth muscle cells. This may increase vascular resistance, particularly in the microcirculation, and ultimately lead to elevated blood pressure [34]. Additionally, studies have shown that the TyG index, a marker combining glucose and triglycerides, is positively associated with the future risk of ischemic heart disease [35]. The above analysis suggests that glucose may serve as a bridge between multiple diseases. Notably, in our study, the association between glucose, WMH, and NCDs was not influenced by a diabetes diagnosis.

It is well known that reducing saturated fatty acids and increasing unsaturated fatty acids in the diet are strategies for lowering cardiovascular disease risk [36]. We observed an upregulation of the saturated fatty acid/total fatty acid (SFA/TFA%) in the hypertension group, which was significantly positively correlated with WMH volume. The linoleic acid/total fatty acid (LA/TFA%) was significantly downregulated in hypertension and was identified as a protective factor against WMH. This is consistent with previous research findings [37, 38, 39]. Further analysis revealed that the LA/TFA%, the percentage of cholesteryl ester in total lipids (CE/TL) within large LDL particles, and the percentage of cholesterol in total lipids (C/TL) within large LDL particles partially mediated the effect of hypertension on WMH volume. Research has found that small LDL particles have a greater ability to penetrate the arterial intima, are more prone to precipitation and oxidation, and have a stronger atherogenic potential, making them a significant risk factor [40]. Large LDL particles are considered ‘good LDL,’ responsible for normal cholesterol transport. In this study, the percentage of cholesteryl ester and cholesterol in total lipids within large LDL particles was reduced in individuals with hypertension and negatively correlated with WMH volume. Combined with the earlier discussion on LA, the mediation analysis suggests that the reduction of hypertension‐associated LA, cholesteryl ester, and cholesterol in large LDL particles contributes to the increase in WMH volume. Additionally, the percentage of phospholipids in total lipids (PL/TL) within large high‐density lipoprotein (HDL) particles fully mediated the effect of hypertension combined with CIHD on WMH volume. Our study showed that PL/TL (L‐HDL) was elevated in individuals with hypertension combined with CIHD and positively correlated with WMH volume. Traditionally, HDL has been considered the ‘good protein.’ However, recent perspectives suggest that the size of HDL particles leads to different pathological effects. A study from the UK Biobank found that larger HDL particles are risk factors for cardiovascular and all‐cause mortality [41]. Another study, which included multiple cohorts, found that large HDL particles are a risk predictor for heart failure [42]. Therefore, the PL/TL (L‐HDL) associated with hypertension combined with CIHD may involve mechanisms that damage the microcirculation, leading to long‐term increases in WMH volume. In summary, these metabolites serve as a bridge linking NCDs and WMH. Given the risks of stroke and dementia associated with WMH, along with the complex burden of multimorbidity, these metabolites may hold the key to addressing multiple challenges.

However, there are certain limitations to this study. First, the inclusion of individuals with WMH at the first imaging assessment has led to selection bias in the retrospective analysis. Second, this study mainly involved participants from the UK Biobank, the vast majority of whom are white, which may limit the generalizability of the findings to other populations and regions. Lastly, there is currently no standardized list of NCDs, with researchers often selecting common diseases or diseases known to have a significant impact on the research objectives, which may result in omissions. Clearly, the concept and types of NCDs are broad and extensive, making it challenging for most studies to encompass all disease categories.

## 5 Conclusions

In summary, this study elucidates the association between WMH and NCDs, showing that several common NCDs significantly increase WMH volume, with NCD‐associated plasma metabolites mediating this risk. This provides new insights into preventing WMH progression in individuals.

## Author Contributions

N.W., B.Q., W.C., Y.P., and Y.W. were involved in the conception of the work. N.W. extracted the data. N.W. and B.Q. contributed to the design, analysis, and interpretation of data for the article. N.W. wrote the first draft of the manuscript. B.Q., Y.P., and W.C. participated in the critical evaluation of the article. Y.W. provided funding support.

## Ethics Statement

The study ethics of the UK Biobank have been approved by the National Health and Social Care Information Governance and the NHS North West Multi‐Center Research Ethics Committee. All participants provided informed consent via electronic signature at the time of recruitment. This study was conducted under UK Biobank application number 135114.

## Conflicts of Interest

The authors declare no conflicts of interest.

## Supporting information


**Table S1.** Exclusion diagnoses based on ICD‐10.
**Table S2.** Metabolite information measured by UK Biobank.
**Table S3.** NCDs selected based on ICD‐10.
**Table S4.** Demographic characteristics and NCDs stratified by WMH volume quartiles.
**Table S5.** List of abbreviations for selected relevant metabolite indicators.
**Figure S1.** Screening of baseline NCD‐related metabolites.
**Figure S2.** NCD‐related metabolites with no significant mediating effect in the relationship between NCDs and WMH volume.

## Data Availability

The data that support the findings of this study are available on request from the corresponding author. The data are not publicly available due to privacy or ethical restrictions.
