# Supplementary material for: Plasma Metabolites Link Non‐Communicable Diseases to Increased White Matter Hyperintensities
Source: CNS Neurosci Ther. 2025 Jul 28;31(7):e70507. doi: 10.1111/cns.70507 (PMC12301696; doi:10.1111/cns.70507)
Supplement: Supplementary file 1 — Table S1. Exclusion diagnoses based on ICD‐10. Table S2. Metabolite information measured by UK Biobank. Table S3. NCDs selected based on ICD‐10. Table S4. Demographic characteristics and NCDs stratified by WMH volume quartiles. Table S5. List of abbreviations for selected relevant metabolite indicators. Figure S1. Screening of baseline NCD‐related metabolites. Figure S2. NCD‐related metabolites with no significant mediating effect in the relationship between NCDs and WMH volume. [file CNS-31-e70507-s001.pdf]

Supplementary Material

TABLE S1 Exclusion diagnoses based on ICD-10..1

TABLE S2 Metabolite information measured by UK Biobank...2

TABLE S3 NCDs selected based on ICD-10...8

TABLE S4 Demographic characteristics and NCDs stratified by WMH volume quartiles............9

TABLE S5 List of abbreviations for selected relevant metabolite indicators...............................10

FIGURE S1 Screening of baseline NCD-related Metabolites 12

FIGURE S2 NCD-related metabolites with no significant mediating effect in the relationship between NCDs and WMH volume..13

TABLE S1 Exclusion diagnoses based on ICD-10

Diagnosis	ICD-10	Field ID in UK Biobank
Hydrocephalus	G91	
Malignant neoplasm of brain	C71	
Intracranial abscess and granuloma	G06.0	
Encephalitis	G04/G05	
Systemic lupus erythematosus	M32	
Ischaemic stroke	/	42008
Demyelinating diseases of the central nervous system	G35/G36/G37	

ICD-10: International Classification of Diseases, 10th Revision

TABLE S2 Metabolite information measured by UK Biobank

Num	Metabolites	Unites
001	Total Cholesterol	mmol/l
002	Total Cholesterol Minus HDL-C	mmol/l
003	Remnant Cholesterol (Non-HDL, Non-LDL-Cholesterol)	mmol/l
004	VLDL Cholesterol	mmol/l
005	Clinical LDL Cholesterol	mmol/l
006	LDL Cholesterol	mmol/l
007	HDL Cholesterol	mmol/l
008	Total Triglycerides	mmol/l
009	Triglycerides in VLDL	mmol/l
010	Triglycerides in LDL	mmol/l
011	Triglycerides in HDL	mmol/l
012	Total Phospholipids in Lipoprotein Particles	mmol/l
013	Phospholipids in VLDL	mmol/l
014	Phospholipids in LDL	mmol/l
015	Phospholipids in HDL	mmol/l
016	Total Esterified Cholesterol	mmol/l
017	Cholesteryl Esters in VLDL	mmol/l
018	Cholesteryl Esters in LDL	mmol/l
019	Cholesteryl Esters in HDL	mmol/l
020	Total Free Cholesterol	mmol/l
021	Free Cholesterol in VLDL	mmol/l
022	Free Cholesterol in LDL	mmol/l
023	Free Cholesterol in HDL	mmol/l
024	Total Lipids in Lipoprotein Particles	mmol/l
025	Total Lipids in VLDL	mmol/l
026	Total Lipids in LDL	mmol/l
027	Total Lipids in HDL	mmol/l
028	Total Concentration of Lipoprotein Particles	mmol/
029	Concentration of VLDL Particles	mmol/l
030	Concentration of LDL Particles	mmol/l
031	Concentration of HDL Particles	mmol/l
032	Average Diameter for VLDL Particles	nm
033	Average Diameter for LDL Particles	nm
034	Average Diameter for HDL Particles	nm
035	Phosphoglycerides	mmol/l
036	Total Cholines	mmol/l
037	Phosphatidylcholines	
038	Sphingomyelins	
039	Apolipoprotein B	g/l
040	Apolipoprotein A1	
041	Total Fatty Acids	mmol/l

042	Degree of Unsaturation	degree
043	Omega-3 Fatty Acids	mmol/l
044	Omega-6 Fatty Acids	mmol/l
045	Polyunsaturated Fatty Acids	mmol/l
046	Monounsaturated Fatty Acids	mmol/l
047	Saturated Fatty Acids	mmol/l
048	Linoleic Acid	mmol/l
049	Docosahexaenoic Acid	mmol/l
050	Alanine	mmol/l
051	Glutamine	mmol/l
052	Glycine	mmol/l
053	Histidine	mmol/l
054	Total Concentration of Branched-Chain Amino Acids (Leucine + Isoleucine + Valine)	mmol/l
055	Isoleucine	mmol/l
056	Isoleucine	mmol/l
057	Valine	mmol/l
058	Phenylalanine	mmol/l
059	Tyrosine	mmol/l
060	Glucose	mmol/l
061	Lactate	mmol/l
062	Pyruvate	mmol/l
063	Citrate	mmol/l
064	3-Hydroxybutyrate	mmol/l
065	Acetate	mmol/l
066	Acetoacetate	mmol/l
067	Acetone	mmol/l
068	Creatinine	mmol/l
069	Albumin	g/l
070	Glycoprotein Acetyls	mmol/l
071	Concentration of Chylomicrons and Extremely Large VLDL Particles	mmol/l
072	Total Lipids in Chylomicrons and Extremely Large VLDL	mmol/l
073	Phospholipids in Chylomicrons and Extremely Large VLDL	mmol/l
074	Cholesterol in Chylomicrons and Extremely Large VLDL	mmol/l
075	Cholesteryl Esters in Chylomicrons and Extremely Large VLDL	mmol/l
076	Free Cholesterol in Chylomicrons and Extremely Large VLDL	mmol/l
077	Triglycerides in Chylomicrons and Extremely Large VLDL	mmol/l
078	Concentration of Very Large VLDL Particles	mmol/l
079	Total Lipids in Very Large VLDL	mmol/l
080	Phospholipids in Very Large VLDL	mmol/l
081	Cholesterol in Very Large VLDL	mmol/l
082	Cholesteryl Esters in Very Large VLDL	mmol/l
083	Free Cholesterol in Very Large VLDL	mmol/l

084	Triglycerides in Very Large VLDL	mmol/l
085	Concentration of Large VLDL Particles	mmol/l
086	Total Lipids in Large VLDL	mmol/l
087	Phospholipids in Large VLDL	mmol/l
088	Cholesterol in Large VLDL	mmol/l
089	Cholesteryl Esters in Large VLDL	mmol/l
090	Free Cholesterol in Large VLDL	mmol/l
091	Triglycerides in Large VLDL	mmol/l
092	Concentration of Medium VLDL Particles	mmol/l
093	Total Lipids in Medium VLDL	mmol/l
094	Phospholipids in Medium VLDL	mmol/l
095	Cholesterol in Medium VLDL	mmol/l
096	Cholesteryl Esters in Medium VLDL	mmol/l
097	Free Cholesterol in Medium VLDL	mmol/l
098	Triglycerides in Medium VLDL	mmol/l
099	Concentration of Small VLDL Particles	mmol/l
100	Total Lipids in Small VLDL	mmol/l
101	Phospholipids in Small VLDL	mmol/l
102	Cholesterol in Small VLDL	mmol/l
103	Cholesteryl Esters in Small VLDL	mmol/l
104	Free Cholesterol in Small VLDL	mmol/l
105	Triglycerides in Small VLDL	mmol/l
106	Concentration of Very Small VLDL Particles	mmol/l
107	Total Lipids in Very Small VLDL	mmol/l
108	Phospholipids in Very Small VLDL	mmol/l
109	Cholesterol in Very Small VLDL	mmol/l
110	Cholesteryl Esters in Very Small VLDL	mmol/l
111	Free Cholesterol in Very Small VLDL	mmol/l
112	Triglycerides in Very Small VLDL	mmol/l
113	Concentration of IDL Particles	mmol/l
114	Total Lipids in IDL	mmol/l
115	Phospholipids in IDL	mmol/l
116	Cholesterol in IDL	mmol/l
117	Cholesteryl Esters in IDL	mmol/l
118	Free Cholesterol in IDL	mmol/l
119	Triglycerides in IDL	mmol/l
120	Concentration of Large LDL Particles	mmol/l
121	Total Lipids in Large LDL	mmol/l
122	Phospholipids in Large LDL	mmol/l
123	Cholesterol in Large LDL	mmol/l
124	Cholesteryl Esters in Large LDL	mmol/l
125	Free Cholesterol in Large LDL	mmol/l
126	Triglycerides in Large LDL	mmol/l

127	Concentration of Medium LDL Particles	mmol/l
128	Total Lipids in Medium LDL	mmol/l
129	Phospholipids in Medium LDL	mmol/l
130	Cholesterol in Medium LDL	mmol/l
131	Cholesteryl Esters in Medium LDL	mmol/l
132	Free Cholesterol in Medium LDL	mmol/l
133	Triglycerides in Medium LDL	mmol/l
134	Concentration of Small LDL Particles	mmol/l
135	Total Lipids in Small LDL	mmol/l
136	Phospholipids in Small LDL	mmol/l
137	Cholesterol in Small LDL	mmol/l
138	Cholesteryl Esters in Small LDL	mmol/l
139	Free Cholesterol in Small LDL	mmol/l
140	Triglycerides in Small LDL	mmol/l
141	Concentration of Very Large HDL Particles	mmol/l
142	Total Lipids in Very Large HDL	mmol/l
143	Phospholipids in Very Large HDL	mmol/l
144	Cholesterol in Very Large HDL	mmol/l
145	Cholesteryl Esters in Very Large HDL	mmol/l
146	Free Cholesterol in Very Large HDL	mmol/l
147	Triglycerides in Very Large HDL	mmol/l
148	Concentration of Large HDL Particles	mmol/l
149	Total Lipids in Large HDL	mmol/l
150	Phospholipids in Large HDL	mmol/l
151	Cholesterol in Large HDL	mmol/l
152	Cholesteryl Esters in Large HDL	mmol/l
153	Free Cholesterol in Large HDL	mmol/l
154	Triglycerides in Large HDL	mmol/l
155	Concentration of Medium HDL Particles	mmol/l
156	Total Lipids in Medium HDL	mmol/l
157	Phospholipids in Medium HDL	mmol/l
158	Cholesterol in Medium HDL	mmol/l
159	Cholesteryl Esters in Medium HDL	mmol/l
160	Free Cholesterol in Medium HDL	mmol/l
161	Triglycerides in Medium HDL	mmol/l
162	Concentration of Small HDL Particles	mmol/l
163	Total Lipids in Small HDL	mmol/l
164	Phospholipids in Small HDL	mmol/l
165	Cholesterol in Small HDL	mmol/l
166	Cholesteryl Esters in Small HDL	mmol/l
167	Free Cholesterol in Small HDL	mmol/l
168	Triglycerides in Small HDL	mmol/l
169	Triglycerides to Phosphoglycerides ratio	

170	Apolipoprotein B to Apolipoprotein A1 ratio	
171	Omega-3 Fatty Acids to Total Fatty Acids percentage	
172	Omega-6 Fatty Acids to Total Fatty Acids percentage	
173	Polyunsaturated Fatty Acids to Total Fatty Acids percentage	
174	Monounsaturated Fatty Acids to Total Fatty Acids percentage	
175	Saturated Fatty Acids to Total Fatty Acids percentage	
176	Linoleic Acid to Total Fatty Acids percentage	
177	Docosahexaenoic Acid to Total Fatty Acids percentage	
178	Polyunsaturated Fatty Acids to Monounsaturated Fatty Acids ratio	
179	Omega-6 Fatty Acids to Omega-3 Fatty Acids ratio	
180	Phospholipids to Total Lipids in Chylomicrons and Extremely Large VLDL percentage	
181	Cholesterol to Total Lipids in Chylomicrons and Extremely Large VLDL percentage	
182	Cholesteryl Esters to Total Lipids in Chylomicrons and Extremely Large VLDL percentage	
183	Free Cholesterol to Total Lipids in Chylomicrons and Extremely Large VLDL percentage	
184	Triglycerides to Total Lipids in Chylomicrons and Extremely Large VLDL percentage	
185	Phospholipids to Total Lipids in Very Large VLDL percentage	
186	Cholesterol to Total Lipids in Very Large VLDL percentage	
187	Cholesteryl Esters to Total Lipids in Very Large VLDL percentage	
188	Free Cholesterol to Total Lipids in Very Large VLDL percentage	
189	Triglycerides to Total Lipids in Very Large VLDL percentage	
190	Phospholipids to Total Lipids in Large VLDL percentage	
191	Cholesterol to Total Lipids in Large VLDL percentage	
192	Cholesteryl Esters to Total Lipids in Large VLDL percentage	
193	Free Cholesterol to Total Lipids in Large VLDL percentage	
194	Triglycerides to Total Lipids in Large VLDL percentage	
195	Phospholipids to Total Lipids in Medium VLDL percentage	
196	Cholesterol to Total Lipids in Medium VLDL percentage	
197	Cholesteryl Esters to Total Lipids in Medium VLDL percentage	
198	Free Cholesterol to Total Lipids in Medium VLDL percentage	
199	Triglycerides to Total Lipids in Medium VLDL percentage	
200	Phospholipids to Total Lipids in Small VLDL percentage	
201	Cholesterol to Total Lipids in Small VLDL percentage	
202	Cholesteryl Esters to Total Lipids in Small VLDL percentage	
203	Free Cholesterol to Total Lipids in Small VLDL percentage	
204	Triglycerides to Total Lipids in Small VLDL percentage	
205	Phospholipids to Total Lipids in Very Small VLDL percentage	
206	Cholesterol to Total Lipids in Very Small VLDL percentage	
207	Cholesteryl Esters to Total Lipids in Very Small VLDL percentage	

208	Free Cholesterol to Total Lipids in Very Small VLDL percentage	
209	Triglycerides to Total Lipids in Very Small VLDL percentage	
210	Phospholipids to Total Lipids in IDL percentage	
211	Cholesterol to Total Lipids in IDL percentage	
212	Cholesteryl Esters to Total Lipids in IDL percentage	
213	Free Cholesterol to Total Lipids in IDL percentage	
214	Triglycerides to Total Lipids in IDL percentage	
215	Phospholipids to Total Lipids in Large LDL percentage	
216	Cholesterol to Total Lipids in Large LDL percentage	
217	Cholesteryl Esters to Total Lipids in Large LDL percentage	
218	Free Cholesterol to Total Lipids in Large LDL percentage	
219	Triglycerides to Total Lipids in Large LDL percentage	
220	Phospholipids to Total Lipids in Medium LDL percentage	
221	Cholesterol to Total Lipids in Medium LDL percentage	
222	Cholesteryl Esters to Total Lipids in Medium LDL percentage	
223	Free Cholesterol to Total Lipids in Medium LDL percentage	
224	Triglycerides to Total Lipids in Medium LDL percentage	
225	Phospholipids to Total Lipids in Small LDL percentage	
226	Cholesterol to Total Lipids in Small LDL percentage	
227	Cholesteryl Esters to Total Lipids in Small LDL percentage	
228	Free Cholesterol to Total Lipids in Small LDL percentage	
229	Triglycerides to Total Lipids in Small LDL percentage	
230	Phospholipids to Total Lipids in Very Large HDL percentage	
231	Cholesterol to Total Lipids in Very Large HDL percentage	
232	Cholesteryl Esters to Total Lipids in Very Large HDL percentage	
233	Free Cholesterol to Total Lipids in Very Large HDL percentage	
234	Triglycerides to Total Lipids in Very Large HDL percentage	
235	Phospholipids to Total Lipids in Large HDL percentage	
236	Cholesterol to Total Lipids in Large HDL percentage	
237	Cholesteryl Esters to Total Lipids in Large HDL percentage	
238	Free Cholesterol to Total Lipids in Large HDL percentage	
239	Triglycerides to Total Lipids in Large HDL percentage	
240	Phospholipids to Total Lipids in Medium HDL percentage	
241	Cholesterol to Total Lipids in Medium HDL percentage	
242	Cholesteryl Esters to Total Lipids in Medium HDL percentage	
243	Free Cholesterol to Total Lipids in Medium HDL percentage	
244	Triglycerides to Total Lipids in Medium HDL percentage	
245	Phospholipids to Total Lipids in Small HDL percentage	
246	Cholesterol to Total Lipids in Small HDL percentage	
247	Cholesteryl Esters to Total Lipids in Small HDL percentage	
248	Free Cholesterol to Total Lipids in Small HDL percentage	
249	Triglycerides to Total Lipids in Small HDL percentage	

TABLE S3 NCDs selected based on ICD-10

Diagnosis	ICD-10
Hypertension	I10
Heart failure	I50
Atrial fibrillation and flutter	I48
Cardiomyopathy	I42
Chronic ischemic heart disease	I25
Dyslipidemia	E78
Type 2 diabetes mellitus	E11
Hypothyroidism	E03
Hyperthyroidism	E05
Obesity	E66
Chronic gastritis	K29.3/K29.4/K29.5
Constipation	K59.0
Liver failure	K72
Chronic hepatitis	K73
Liver fibrosis and cirrhosis	K74
Fatty liver	K76.0
Intestinal malabsorption	K90
Chronic renal failure	N18
Chronic nephritis	N03
Nephrotic syndrome	N04
Nutritional anemia	D50/D51/D52/D53
Chronic obstructive pulmonary disease	J44
Asthma	J45
Emphysema	J43
Respiratory failure	J96
Nonorganic headache	G43/G44
Obstractive sleep apnea	G47.3
Depressive disorder	F32
Anxiety disorder	F41

NCDs: Non-communicable Diseases; ICD-10: International Classification of Diseases, 10th Revision

TABLE S4 Demographic characteristics and NCDs stratified by WMH volume quartiles

	Q1(N=11,161)	Q2(N=11,154)	Q3(N=11,158)	Q4(N=11,157)	P-value
Male, n (%)	4556 (40.8)	5042 (45.2)	5408 (48.5)	5989 (53.7)	<0.001
Age(year), Mean ±SD	58.94±6.65	62.81±7.00	66.00±6.78	69.40±6.36	<0.001
Ethnicity*, n (%)					<0.001
White	10724 (96.1)	10715 (96.1)	10829 (97.1)	10902 (97.7)	
Non-White	410 (3.7)	411 (3.7)	294 (2.6)	222 (2.0)	
SBP (mmHg), Mean ±SD	134.54±18.31	139.41±19.41	144.01±19.69	149.37±20.26	<0.001
DBP (mmHg), Mean ±SD	77.53±10.33	78.51±10.65	79.44±10.70	80.32±10.90	<0.001
BMI, Mean ±SD	25.90±4.19	26.39±4.36	26.72±4.43	27.04±4.47	<0.001
Smoking status*, n (%)					<0.001
Never	7577 (67.9)	7165 (64.2)	6770 (60.7)	6226 (55.8)	
Previous	3124 (28.0)	3502 (31.4)	3967 (35.6)	4362 (39.1)	
Current	387 (3.5)	392 (3.5)	312 (2.8)	381 (3.4)	
Alcohol drinker status*, n (%)					<0.001
Never	379 (3.4)	323 (2.9)	346 (3.1)	369 (3.3)	
Previous	357 (3.2)	338 (3.0)	388 (3.5)	390 (3.5)	
Current	10370 (92.9)	10425 (93.5)	10349 (92.7)	10267 (92.0)	
Sleep duration (hours/day), Mean ±SD	7.07±1.04	7.11±1.07	7.16±1.09	7.20±1.14	<0.001
Physical activity (minutes/week), Median (IQR)	2142.0(1110.0-3776.5)	2212.5(1150.0-3944.0)	2232.0(1152.0-3972.0)	2196.0(1113.0-3906.0)	0.034
Household income (pounds)*, n (%)					<0.001
Less than 18,000	972 (8.7)	1157 (10.4)	1311 (11.7)	1444 (12.9)	
18,000 to 30,999	2132 (19.1)	2613 (23.4)	2944 (26.4)	3162 (28.3)	
31,000 to 51,999	3054 (27.4)	3093 (27.7)	3039 (27.2)	3031 (27.2)	
52,000 to 100,000	3010 (27.0)	2418 (21.7)	2021 (18.1)	1726 (15.5)	
Greater than 100,000	1116 (10.0)	806 (7.2)	621 (5.6)	413 (3.7)	
Education*, n (%)					<0.001
No college or university degree	5106 (45.7)	5179 (46.4)	5162 (46.3)	5168 (46.3)	
College or university degree	5505 (49.3)	5199 (46.6)	4949 (44.4)	4747 (42.5)	
Number of NCDs, n (%)					<0.001
0 NCDs	8741 (78.3)	8109 (72.7)	7456 (66.8)	6495 (58.2)	
1 NCDs	1582 (14.2)	1796 (16.1)	1977 (17.7)	2259 (20.2)	
2 NCDs	522 (4.7)	708 (6.3)	942 (8.4)	1248 (11.2)	
3 NCDs	214 (1.9)	334 (3.0)	450 (4.0)	632 (5.7)	
4 NCDs	72 (0.6)	120 (1.1)	204 (1.8)	303 (2.7)	
≥ 5 NCDs	30 (0.3)	87 (0.8)	129 (1.2)	220 (2.0)	
Circulation system disease, n (%)	913 (8.2)	1494 (13.4)	2149 (19.3)	3165 (28.4)	<0.001
Metabolic and endocrine disease, n (%)	801 (7.2)	1136 (10.2)	1558 (14.0)	2012 (18.0)	<0.001
Digestive system disease, n (%)	339 (3.0)	401 (3.6)	470 (4.2)	508 (4.6)	<0.001
Urinary system disease, n (%)	42 (0.4)	60 (0.5)	90 (0.8)	158 (1.4)	<0.001
Hematologic disease, n (%)	207 (1.9)	217 (1.9)	183 (1.6)	259 (2.3)	0.003
Respiratory disease, n (%)	620 (5.6)	660 (5.9)	717 (6.4)	839 (7.5)	<0.001
Neurological and psychiatric disease, n (%)	449 (4.0)	532 (4.8)	570 (5.1)	621 (5.6)	<0.001

NCDs: Non-communicable diseases; WMH: White matter hyperintensities; SBP: Systolic blood pressure; DBP: Diastolic blood pressure; BMI: Body mass index.

* The results for "Do not know", "Prefer not to answer" and "Missing values" have been omitted.

TABLE S5 List of abbreviations for selected relevant metabolite indicators

**Metabolite indicators**	**Abbreviations**
Total Concentration of Branched-Chain Amino Acids (Leucine + Isoleucine + Valine)	BCAA
Triglycerides in HDL	HDL-TG
Triglycerides in Medium HDL	M-HDL-TG
Triglycerides in Small HDL	S-HDL-TG
Triglycerides in Small VLDL	S-VLDL-TG
Triglycerides in Very Small VLDL	VS-VLDL-TG
Acetoacetate	Acetoacetate
Cholesterol to Total Lipids in IDL percentage	C/TL (IDL)%
Cholesterol to Total Lipids in Medium LDL percentage	C/TL (M-LDL) %
Cholesteryl Esters to Total Lipids in IDL percentage	CE/TL (IDL)%
Isoleucine	Isoleucine
Omega-6 Fatty Acids to Omega-3 Fatty Acids ratio	Omega-6/Omega-3
Cholesterol to Total Lipids in Large LDL percentage	C/TL (L-LDL) %
Phospholipids in Small LDL	PL (S-LDL)
Phospholipids to Total Lipids in Medium LDL percentage	PL/TL (M-LDL) %
Phospholipids to Total Lipids in Very Large VLDL percentage	PL/TL (VL-VLDL) %
Phospholipids to Total Lipids in Very Small VLDL percentage	PL/TL (VS-VLDL) %
Saturated Fatty Acids to Total Fatty Acids percentage	SFA/TFA%
Triglycerides to Total Lipids in Chylomicrons and Extremely Large VLDL percentage	TG/TL (Chy+XL-VLDL) %
Valine	Valine
Acetate	Acetate
Acetone	Acetone
Albumin	Albumin
Cholesteryl Esters to Total Lipids in Chylomicrons and Extremely Large VLDL percentage	CE/TL (Chy+XL-VLDL) %
Cholesteryl Esters to Total Lipids in Large LDL percentage	CE/TL (L-LDL) %
Cholesteryl Esters to Total Lipids in Very Large HDL percentage	CE/TL (VL-HDL) %
Concentration of Small LDL Particles	S-LDL Particles
Concentration of Very Large HDL Particles	VL-HDL Particles
Docosahexaenoic Acid	Docosahexaenoic Acid
Free Cholesterol to Total Lipids in Large VLDL percentage	FC/TL (L-VLDL) %
Glucose	Glucose
Glutamine	Glutamine
Glycoprotein Acetyls	Glycoprotein Acetyls
Linoleic Acid to Total Fatty Acids percentage	LA/TFA%
Omega-3 Fatty Acids	Omega-3 Fatty Acids
Cholesterol in Very Large HDL	VL-HDL-C
Cholesteryl Esters in Medium VLDL	M-VLDL-CE
Free Cholesterol to Total Lipids in Medium VLDL percentage	FC/TL (M-VLDL) %
Phospholipids to Total Lipids in Large HDL percentage	PL/TL (L-HDL) %
Phospholipids to Total Lipids in Medium VLDL percentage	PL/TL (M-VLDL) %
Pyruvate	Pyruvate

Total Lipids in IDL	IDL-TL
Triglycerides to Total Lipids in Medium VLDL percentage	TG/TL (M-VLDL) %
Triglycerides to Total Lipids in Very Small VLDL percentage	TG/TL (VS-VLDL) %
Cholesterol in IDL	IDL-C
Cholesterol in Very Small VLDL	VS-VLDL-C
Cholesterol to Total Lipids in Medium VLDL percentage	C/TL (M-VLDL) %
Cholesterol to Total Lipids in Very Small VLDL percentage	C/TL (VS-VLDL) %
Cholesteryl Esters in Very Large HDL	VL-HDL-CE
Cholesteryl Esters in Very Small VLDL	VS-VLDL-CE
Cholesteryl Esters to Total Lipids in Very Small VLDL percentage	CE/TL (VS-VLDL) %
Concentration of IDL Particles	IDL Particles
Creatinine	Creatinine
Free Cholesterol in IDL	IDL-FC
Free Cholesterol in Small LDL	S-LDL-FC
Free Cholesterol in Very Large HDL	VL-HDL-FC
Free Cholesterol to Total Lipids in IDL percentage	FC/TL (IDL) %
Free Cholesterol to Total Lipids in Small HDL percentage	FC/TL (S-HDL) %
Free Cholesterol to Total Lipids in Very Small VLDL percentage	FC/TL (VS-VLDL) %
Glucose-lactate	Glucose-lactate
Phospholipids in IDL	IDL-PL
Phenylalanine	Phenylalanine
Free Cholesterol to Total Lipids in Large HDL percentage	FC/TL (L-HDL) %

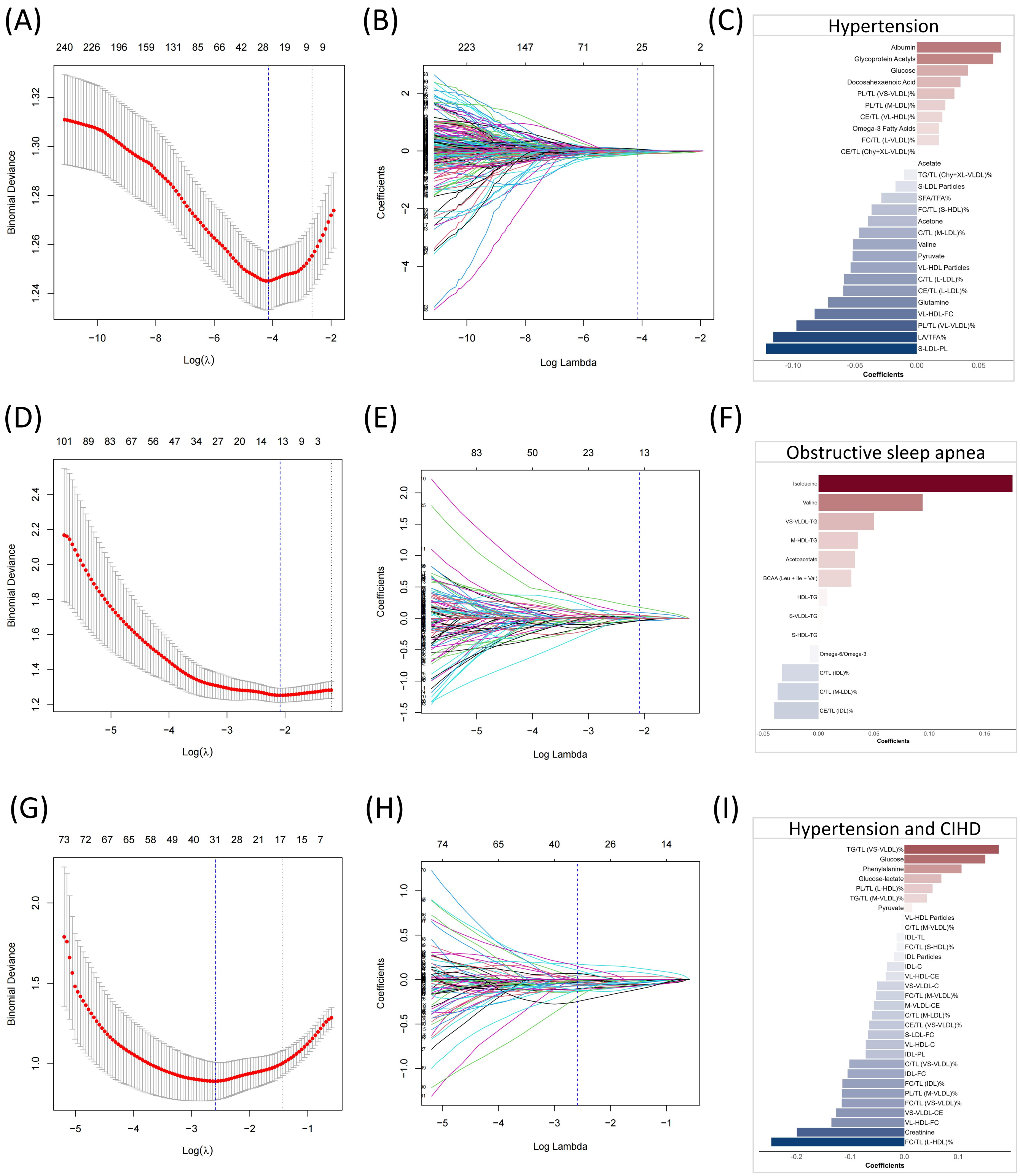

FIGURE S1 Screening of baseline NCD-related Metabolites. (A, B), (D, E), (G, H) represent the process of selecting metabolites related to hypertension, OSA, and hypertension combined with CIHD using the optimal regularization parameter in elastic net regression. (C, F and I) show the coefficients of the selected metabolites in the regression model for hypertension, OSA, and hypertension combined with CIHD, respectively. OSA: Obstructive sleep apnea; CIHD: Chronic ischemic heart disease; and abbreviations of all metabolite indicators are listed in Table S5

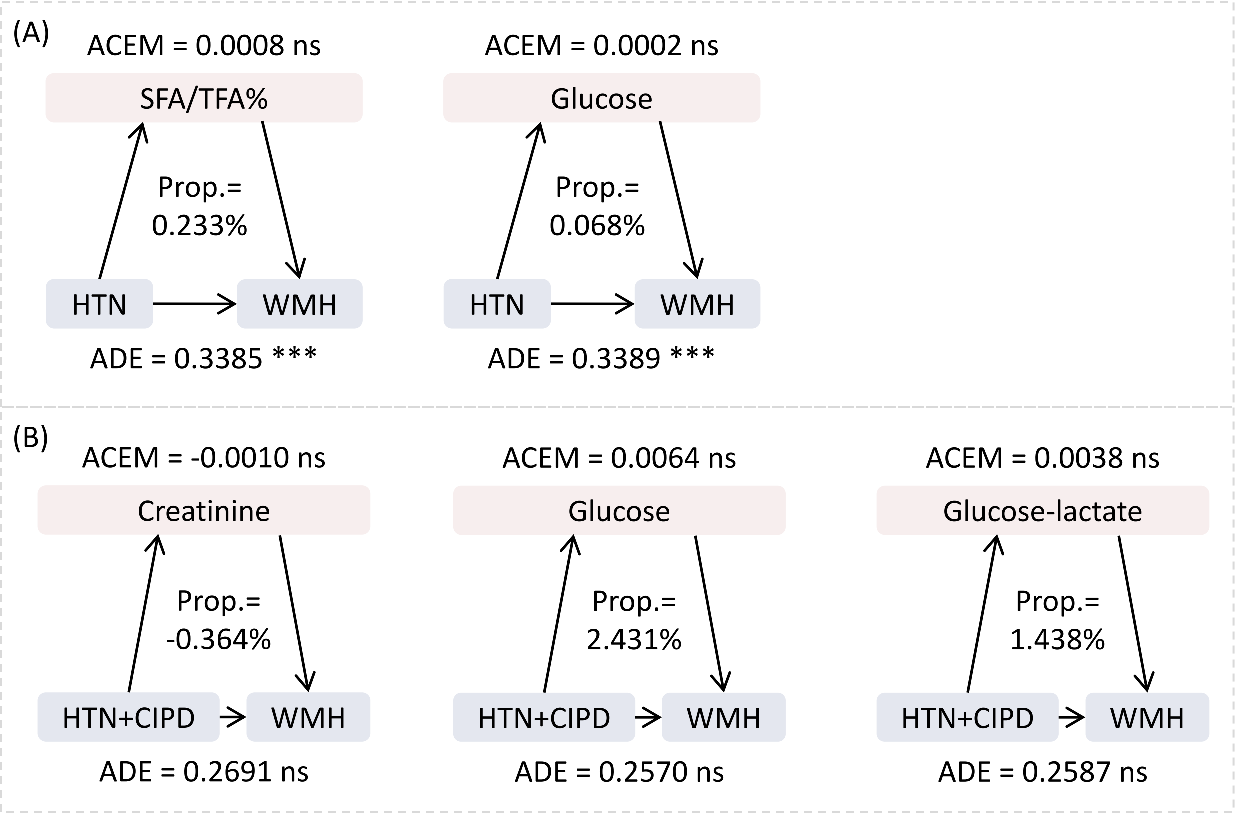

FIGURE S2 NCD-related metabolites with no significant mediating effect in the relationship between NCDs and WMH volume. (A) Mediating effect analysis of hypertension-related metabolites in the association between hypertension and WMH volume. (B) Mediating effect analysis of metabolites associated with hypertension combined with CIHD in the association between hypertension with CIHD and WMH volume. HTN: Hypertension; CIHD: Chronic ischemic heart disease; SFA/TFA:Saturated fatty acids to total fatty acids; ACME: Average causal mediation effect; ADE: Average direct effect. *** $p < 0.001$ ** $p < 0.01$ * $p < 0.05$ ns $p \geq 0.05$
